# Bioconjugation of COL1 protein on liquid-like solid surfaces to study tumor invasion dynamics

**DOI:** 10.1116/6.0002083

**Published:** 2023-03-10

**Authors:** D. T. Nguyen, D. I. Pedro, A. Pepe, J. G. Rosa, J. I. Bowman, L. Trachsel, G. R. Golde, I. Suzuki, J. M. Lavrador, N. T. Y. Nguyen, M. A. Kis, R. A. Smolchek, N. Diodati, R. Liu, S. R. Phillpot, A. R. Webber, P. Castillo, E. J. Sayour, B. S. Sumerlin, W. G. Sawyer

**Affiliations:** 1 Department of Mechanical and Aerospace Engineering, Herbert Wertheim College of Engineering, College of Medicine University of Florida, Gainesville, Florida 3261; 2 Department of Chemistry, College of Liberal Arts and Sciences, College of Medicine University of Florida, Gainesville, Florida 3261; 3 Department of Surgery, College of Medicine University of Florida, Gainesville, Florida 3261; 4 Department of Materials Science and Engineering Herbert Wertheim College of Engineering, College of Medicine University of Florida, Gainesville, Florida 3261; 5 Department of Pediatrics, College of Medicine University of Florida, Gainesville, Florida 3261; 6 Department of Anatomy and Cell Biology, College of Medicine University of Florida, Gainesville, Florida 3261

## Abstract

Tumor invasion is likely driven by the product of intrinsic and extrinsic stresses, reduced intercellular adhesion, and reciprocal interactions between the cancer cells and the extracellular matrix (ECM). The ECM is a dynamic material system that is continuously evolving with the tumor microenvironment. Although it is widely reported that cancer cells degrade the ECM to create paths for migration using membrane-bound and soluble enzymes, other nonenzymatic mechanisms of invasion are less studied and not clearly understood. To explore tumor invasion that is independent of enzymatic degradation, we have created an open three-dimensional (3D) microchannel network using a novel bioconjugated liquid-like solid (LLS) medium to mimic both the tortuosity and the permeability of a loose capillary-like network. The LLS is made from an ensemble of soft granular microgels, which provides an accessible platform to investigate the 3D invasion of glioblastoma (GBM) tumor spheroids using *in situ* scanning confocal microscopy. The surface conjugation of the LLS microgels with type 1 collagen (COL1-LLS) enables cell adhesion and migration. In this model, invasive fronts of the GBM microtumor protruded into the proximal interstitial space and may have locally reorganized the surrounding COL1-LLS. Characterization of the invasive paths revealed a super-diffusive behavior of these fronts. Numerical simulations suggest that the interstitial space guided tumor invasion by restricting available paths, and this physical restriction is responsible for the super-diffusive behavior. This study also presents evidence that cancer cells utilize anchorage-dependent migration to explore their surroundings, and geometrical cues guide 3D tumor invasion along the accessible paths independent of proteolytic ability.

## I. INTRODUCTION

Cancer progression can be characterized by the aggressive proliferation and chaotic activities of cells that disrupt the tissue architecture, thereby leading to improper organ function.^1,2^ Fundamentally, cancer invasion is a three-dimensional (3D) process. In some cancers, the process stimulates the initiation of metastasis to distant organs. It is widely suggested that tumor invasion is triggered by multifactorial determinants including accumulated mutations,^3^ growth-induced competition for space,^4^ deprivation of nutrients and oxygen,^5^ reduction of intercellular adhesion,^6^ and increasing interactions with the surrounding extracellular matrix (ECM);^7^ however, detailed mechanism behind each determinant has remained elusive. Efforts in computational modeling suggest that invasion is an emergent property of the collective cell response to environmental cues.^8^ Additionally, the reduction in intercellular adhesion, caused by genomic instability and various stress factors, may further promote cancer cells to increasingly interact with their physical surroundings. Such interactions reciprocally stimulate cell-ECM engagement and cell migration out of the tumor mass. Once initiated, the invasive cancer cells, individually or collectively, penetrate the ECM and spread across the tumor microenvironment (TME).

Cancer cell migration is thought to be regulated not only by cell-generated contractility and proteolytic activity but also by extrinsic factors such as the presence of adhesion molecules,^6^ soluble factors (e.g., chemotaxis), and topographical and mechanical cues (e.g, durotaxis).^7,9–11^ Due to the dense network of fibrous ECM proteins, early pioneering cancer cells must degrade the ECM via membrane-bound and secreted enzymes (e.g., Matrix metalloproteinases—MMPs) to create space for growth and migration.^12–14^ Existing 3D tumor invasion models employ enzymatically degradable hydrogel platforms (e.g., Matrigel^™^ or collagen) to emulate the migratory processes. Furthermore, contact guidance, imposed by the topographical features of a substrate, has been well-known to direct cell migration.^11,15^ For instance, cancer cells, both individually and collectively, have been reported to align their migration with collagen fibers to facilitate local invasion.^16^ Nevertheless, cancer cell migration is quite dynamic and constantly evolving. While cancer invasion is critically dependent on proteolytic enzymes, an increasing number of studies have reported the dynamic shift to nonproteolytic modes of invasion.^17–21^ Cancer cells can squeeze through accessible pores of collagen networks or confined microchannels independent of proteolytic activities by adopting amoeboid migration.^21–25^ Thus, cancer cells can dynamically adapt various modes of migration in response to heterogeneous environmental cues,^17,18^ yet the underlying mechanism is not clearly demonstrated. The continued development of new biomaterials and biointerfaces for studying tumor invasion provides additional insight into these mechanisms from different perspectives.

Many aspects of tumor invasion are modulated through interactions with the ECM, which represents a complex 3D-compliant network of biological interfaces (e.g., collagens, fibronectin, elastin, and proteoglycans).^26–28^ The ECM is an aqueous, tissue-specific network formed from proteins and polysaccharides that is strikingly heterogeneous. Proteoglycans and fibrous proteins are the two main classes of biomacromolecules of the ECM.^26^ Recent advancements in biotechnologies and material science have made progress in developing materials that are recapitulative of *in vivo* ECM to study cancer progression. Multifunctional hydrogels and microgels have emerged as the main classes of materials employed for the development of novel three-dimensional preclinical models.^29–31^ As compared to conventional cell monolayer models, these 3D *in vitro* models of invasion facilitate more realistic tumor architectures and microenvironments that faithfully recapitulate *in vivo* conditions.^32–34^ In previous studies, we demonstrated a liquid-like solid (LLS) platform for the 3D culture of cells and microtissues.^34,35^ The LLS is made from an ensemble of soft aqueous microgels^36^ that provides an accessible platform for *in situ* observations of drug screening and disease pathogenesis. The LLS can be made of high-water-content hydrogel materials^37–40^ including polyethylene glycol (PEG), polyacrylamide, and others. The sizes of these microgels are designed with characteristic interstitial spaces through which cells can crawl and liquid media can be perfused.

Glioblastoma is a particularly aggressive and fast-growing form of brain cancer with an abysmal median survival of under 2 years.^5,41^ A key characteristic of glioblastoma is its invasion and general lack of a defined tumor margin. Postmortem studies have shown that 20%–27% of glioblastomas invade as much as 10 mm,^42,43^ while approximately another 20% show extensive invasion greater than 30 mm,^43^ and a further 8% show grossly disseminated spread.^41,44^ Due to the aggressiveness of the disease, glioblastoma has been widely employed as an *in vitro* model for tumor invasion. In this study, we report on the development of tunable biointerfaces on a 3D LLS medium with a controlled density of ECM biomacromolecules to facilitate the studies of glioblastoma invasion. In particular, we successfully functionalized and conjugated type 1 collagen onto the surface of LLS microgel particles (COL1-LLS) to enable cell adhesion. The interstitial space between the microgels formed randomly interconnected 3D microchannel networks, allowing cancer cell invasion without the need for ECM degradation. The microgel particles, made of polyacrylamide, were sized to facilitate an interstitial space on the order of 7–10 *μ*m to mimic the characteristics of a 3D capillary network.^45^ The microgel particles gravitationally settle to form a solid bed of yield-stress fluid that stably supports the tumors in 3D. The high-water-content LLS medium has a yield stress of less than 10 Pa, allowing for essentially unrestricted tumor growth and expansion. This platform enabled 3D tumor invasion via cell anchorage-dependent migration and geometrical guidance from the physical surroundings.

## II. MATERIALS AND METHODS

### A. Cell culture

High-grade murine glioma cell line Kr158B was kindly provided by Dr. Elias Sayour, MD. The cell line was cultured in a growth medium containing DMEM without sodium pyruvate (Gibco, Billings, MT, USA, 11965-092), 10% fetal bovine serum (FBS) (Sigma-Aldrich, Sigma-Aldrich, St. Louis, MO, USA, F4135), and 1% penicillin/streptomycin (Gibco, Billings, MT, USA, 15140148) in a T75 cell culture flask at 37 °C and 5% CO_2_. For spheroid generation, we employed a perfusion culture method as previously published in Ref. 34. In short, Kr158B cells were enzymatically detached from cell culture flasks and suspended in inert LLS at a density of 10^6^ cells/ml. The cell-LLS mixture was dispensed into each well (200 *μ*l each) of a 24-well Darcy perfusion plate^34^ and cultured at a perfusion flow rate of ∼40 *μ*l/h/well at 37 °C, 5% CO_2_. Cellular spheroids formed within 72 h of culture. When the spheroid diameter reached 400–600 *μ*m in diameter (∼day 7), the samples were harvested for invasion study.

### B. Fabrication of protein-conjugated polyacrylamide LLS microgels

Acrylamide (AAm) monomer and *N*-acryloxysuccinimide (NAS) (at 5 wt. % total monomer concentration) were copolymerized with *N*,*N′*-methylenebisacrylamide crosslinker (BIS) (0.2 wt. %). The polymerization was initiated by ammonium persulfate and catalyzed by tetramethylethylenediamine via free radical polymerization. The polymerized P(AAm-*co*-NAS) hydrogel was mechanically ruptured to create microgel particles. The microgel size was further homogenized to a target mean and standard deviation with a 95% confidence interval via centrifugation. The P(AAm-*co*-NAS) microgels functionalized with NAS enable covalent modification via the ε-amino (NH_2_) group of lysine residues and the terminal NH_2_ groups present in target proteins. For collagen-conjugated LLS (COL1-LLS), 10 *μ*g/ml of type I collagen (MW: 300 kDa, Nutragen, Cat. 5010) or 33.3 nM per every ml of LLS was used during the conjugation process. To prevent hydrolysis of unconjugated NAS groups to acrylic acid, ethanolamine was added postconjugation to form biologically inert hydroxylethyl acrylamide.

Alternatively, AAm monomer 4%w/v, BIS 0.3%w/v, and acrylic acid (AA) 1%w/v hydrogel were prepared via free radical polymerization in *p*H 5.5 MES buffer (0.7 M). The particles were then activated in a solution of EDC and NHS. The activated microgels were conjugated with collagen I in PBS for 2 h and subsequently quenched with ethanolamine.

### C. 3D invasion assay

The COL1-LLS was equilibrated in cell culture media in 37 °C water bath for 30 min prior to the experiment. The solution was then centrifuged at 1000×g for 5 min to tightly consolidate the COL1-LLS. Upon centrifugation, the liquid media was removed leaving the media-infused and consolidated COL1-LLS at the bottom of the tube. Using a prewet 200 *μ*l-wide bore pipette tip, a volume of 100 *μ*l of COL1-LLS was deposited into the respective well of a glass-bottom 96-well plate. The tumor spheroids were then gently positioned into the COL1-LLS gel such that they were optically accessible. The 96-well plate was then centrifuged at 100×g for 5 min at a low deceleration and acceleration setting. Using a 25G syringe needle, 100 *μ*l of media was gently added on top of the well. The plate was then secured in a custom-built stage incubator of a Nikon A1R HD25 confocal microscope for *in situ* time-lapse acquisition.

### D. Immunofluorescence assay

The immunofluorescence (IF) staining protocol has been previously described.^34,35^ In short, cells were fixed in 4.0% formaldehyde (Fisher Scientific, Waltham, MA, USA, BP531-500) in 1× PBS overnight at 4 °C, washed twice, and incubated in PBS for 1 h at room temperature. The samples were then permeabilized in 0.5% Triton X-100 (Sigma-Aldrich, St. Louis, MO, USA, X100-100ML) for 2 h, washed twice, and blocked with 3% bovine serum albumin in PBS for 3 h at room temperature. After blocking, samples were washed three times with PBS and then incubated overnight with conjugated antibodies at 4 °C. The samples were then stained with Invitrogen^™^ Alexa Fluor^™^ 568 Phalloidin (Invitrogen^TM^, Waltham, MA, USA, A12380). After overnight incubation with the antibodies, the samples were washed three times with PBS and counterstained with Hoechst 33342 (Invitrogen^TM^, Waltham, MA, USA, H3570) for 10 min before imaging.

### E. Microscopy

To capture time-lapse images of glioblastoma invasion, cellular spheroids were fluorescently dyed with Cell Tracker Orange CMRA (5 *μ*M) (Thermo Fisher Scientific, Waltham, MA, USA, C34551) for 30 min in growth media at 37°C and 5% CO_2_. Samples were then rinsed with 1× PBS three times. All samples were imaged using a Nikon A1R HD25 confocal microscope equipped with a high-definition Galvano scanner.

## III. RESULTS AND DISCUSSION

**FIG. 1.**
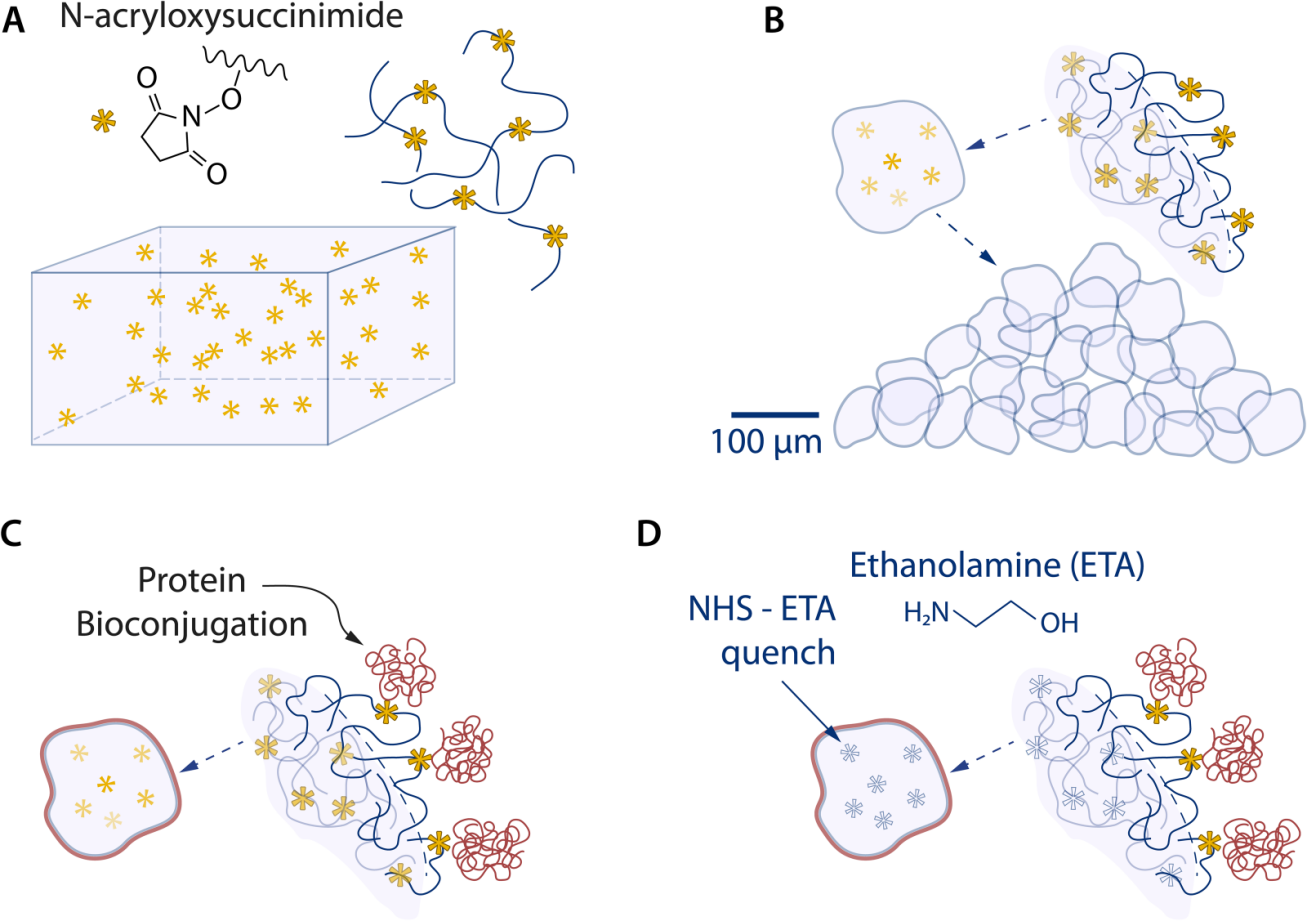
Fabrication of functionalized polyacrylamide microgels. (a) A hydrogel sample is prepared with a functional NHS-acrylate (*N*-Acryloxysuccinimide -NAS) crosslinked into the backbone of the polymer. (b) The microgels are produced after polymerization by means of a mechanical micronization technique yielding microgels that contain the functionalized NHS moieties on the surface and within their interiors. (c) The microgels are conjugated with a type I collagen protein. (d) After the proteins have been conjugated, the microgels are rinsed. Ethanolamine was then added to quench the remaining NHS groups to prevent hydrolysis of unconjugated NAS groups to acrylic acid thereby maintaining charge neutrality of the LLS.

**FIG. 2.**
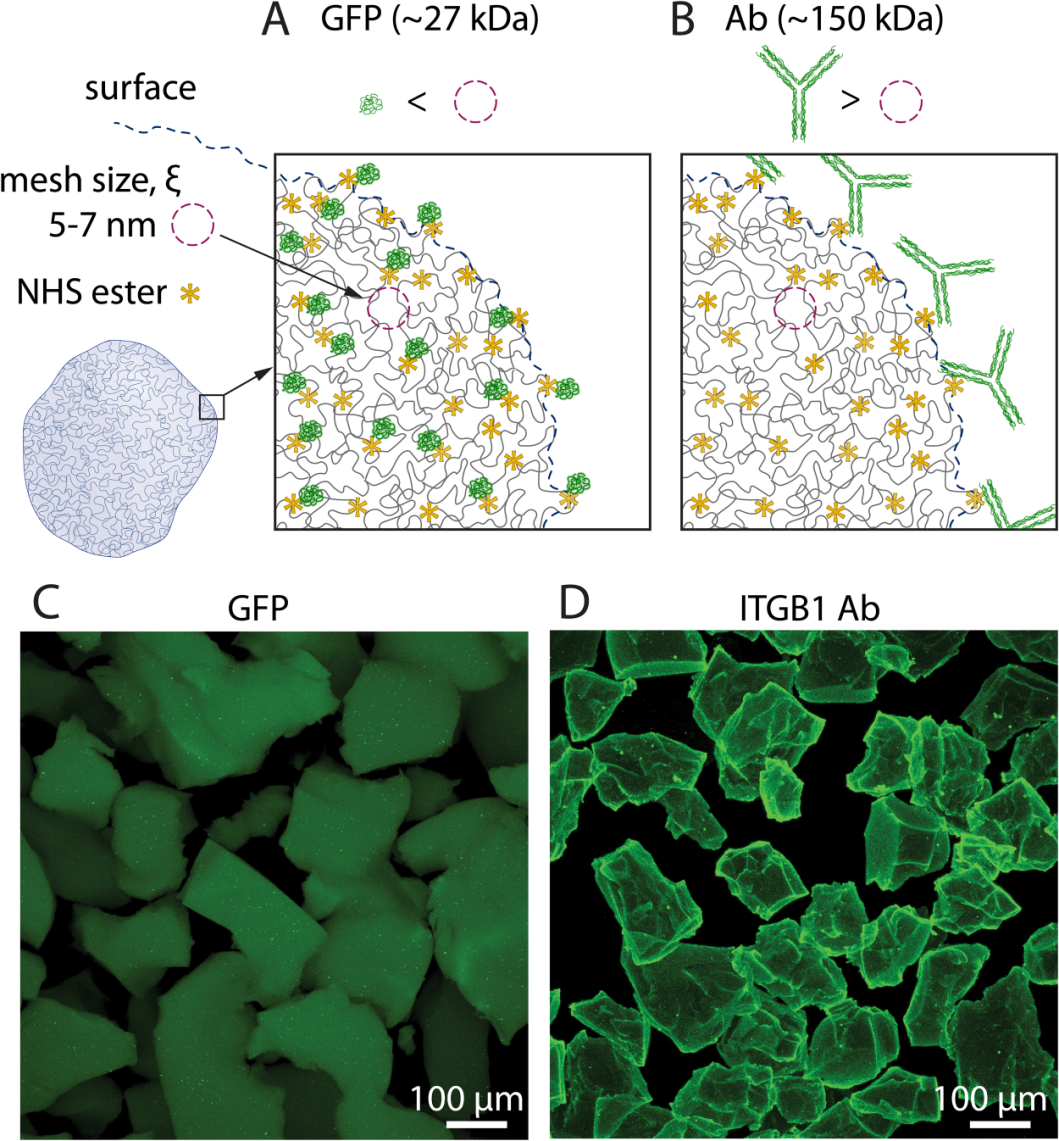
Protein conjugation at the inner vs peripheral region of microgels. (a) and (b) Illustration of the relationship between the size of the target proteins vs the mesh size (ξ) of the polyacrylamide microgels. (a) Small proteins can diffuse through layers of flexible polymer network and react with NHS ester reactive groups distributed on the backbone of the polymer chains of a microgel particle. (b) Proteins or antibodies that are larger than the mesh size of the microgels will locally react at the peripheral region of the particles, thereby selectively coating the surfaces of the microgels. (c) and (d) Confocal microscopy images of protein-conjugated microgels showing the presence of conjugated proteins on two distinct regions: inner region (c) with GFP (50 *μ*g/ml) and a peripheral region (d) with an antibody (1 *μ*g/ml.).

**FIG. 3.**
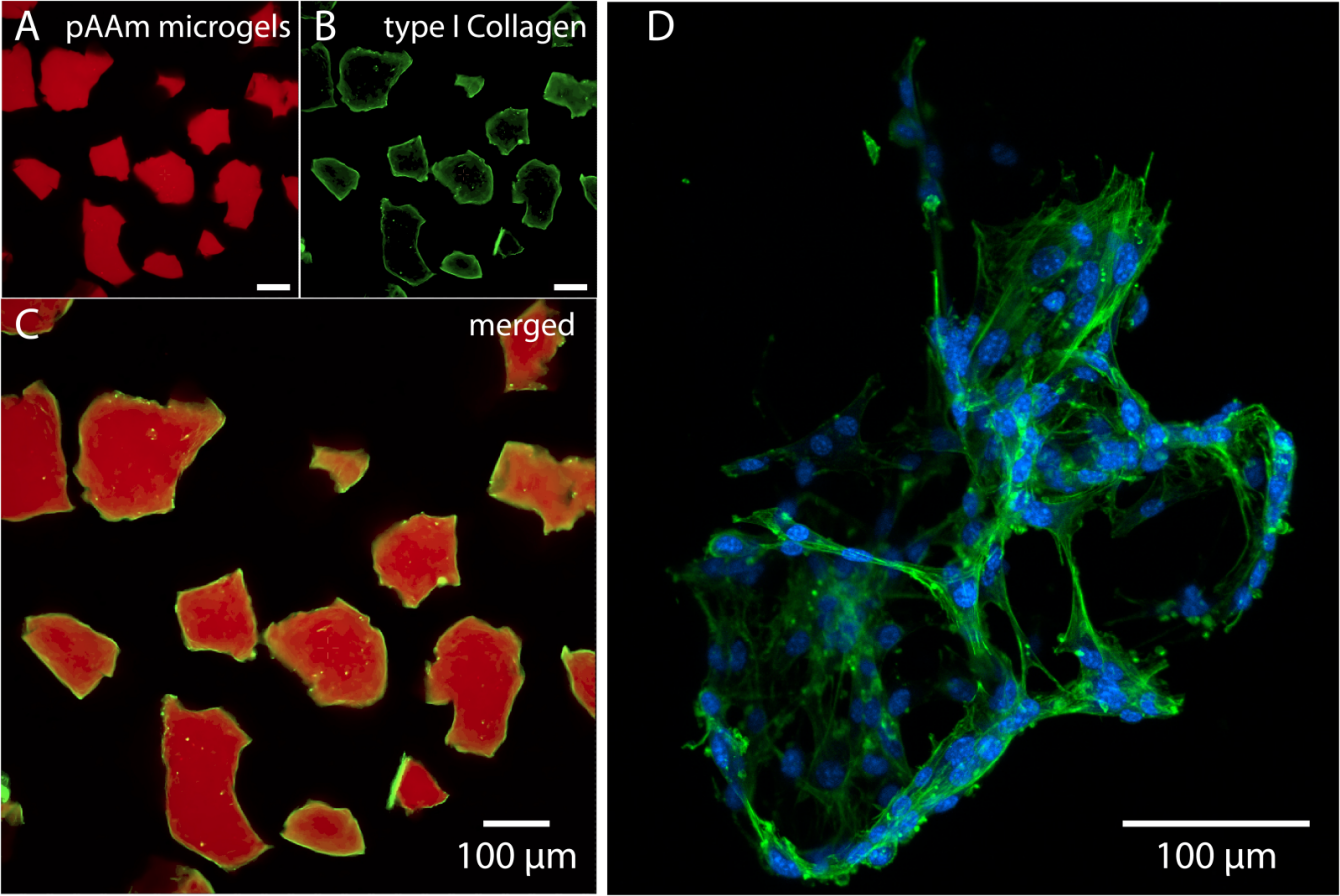
Collagen I conjugated microgels facilitate cellular adhesion. (a) Fluorescent image of polyacrylamide copolymerized with NAS and acryloxyethyl thiocarbamoyl rhodamine B. (b) and (c) Presence of type I collagen conjugated on the surface of the microgels was confirmed by confocal microscopy. (d) A fluorescent image of Kr158B cells adhering onto collagen-conjugated LLS particles indicating the collagen-coated LLS microgels can facilitate cell adhesion. The cells were stained for nuclei (blue) and F-actin (green).

In this study, we developed a protein-conjugated liquid-like solid (LLS) from a polyacrylamide microgel ensemble. We functionalized the microgels by copolymerizing acrylamide monomers and acrylic acid *N*-hydroxysuccinimide esters (*N*-acryloxysuccinimide) with *N*,*N′*-methylenebisacrylamide crosslinker. The bioconjugated microgels were also produced via EDC/NHS coupling. In this case, we copolymerized acrylamide with acrylic acid that were then activated *in situ* via EDC/NHS chemistry to form the pendant activated NHS esters. The free radical polymerization was initiated by ammonium persulfate and catalyzed by a tetramethylethylenediamine (TEMED) reductant. The NHS-polyacrylamide hydrogel was mechanically disintegrated into microgel particles whose surfaces were further conjugated with an extracellular matrix protein to promote cell adhesion (Fig. 1). The amine-reactive NHS esters enabled covalent conjugations with primary amines at the N-terminus and lysine amino acid residues present on all proteins. The major advantage of the EDC/NHS method compared to the one containing *N*-acryloxysuccinimide is the tunability of the *p*H for each step, reducing the risk of hydrolysis and increasing the yield of the bioconjugation reaction. This mechanism allowed for the control of different proteins to be conjugated on LLS microgels, potentiating the recapitulation of tissue-specific ecosystems in vitro via various protein compositions. In this study, type I collagen (COL1: ∼300 kDa molecule^46^ with a radius of gyration R_g_ ∼ 25–36 nm), an abundant ECM protein present in all tissues, was selected as a proof of concept. The surface modification of the microgels and successful conjugation of the proteins were first demonstrated by confocal microscopy (Fig. 2) and the functionality was confirmed by cell attachment and spreading (Fig. 3).

The spatial distribution of the conjugated proteins within the microgel particle could be greatly controlled by the polyacrylamide gel mesh size ( ξ), which is dependent on total monomer content and the ratio of monomers to crosslinkers.^47^ These factors are coupled determinants of the elastic modulus, which has been shown to regulate cell adhesion and migratory behavior.^48^ The polymer mesh size is essentially defined as the average spacing of all polymer chains. This parameter is integral to the mechanical and transport properties of hydrogels including hydraulic permeability,^49^ osmotic pressure,^50^ and elastic modulus.^50,51^ Polyacrylamide mess size, typically 1–10 nm,^52^ is commonly characterized by small-angle x-ray scattering (SAXS).^53^ Polyacrylamide hydrogel mesh size is modulated by the concentration of monomers and crosslinkers.^47,52^ The region of protein conjugation on LLS microgels is highly dependent on the polymer mesh size which is directly associated with elastic modulus. For small proteins (r_g_ < ξ), the conjugation is uniform throughout the entire microgel particle. Proteins with characteristic size (e.g., the radius of gyration) larger than the polymer mesh size (r_g_ > ξ) were conjugated at the peripheral regions of the microgels due to particle exclusion. Therefore, surface modification of the polyacrylamide microgels with conjugated proteins was achieved by modulating mesh size to avoid diffusivity of proteins to the inner region of the gel. In this study, we chose a formulation of 4.8% (w/v) Aam, 0.2% (w/v) BIS, and 0.5% (w/v) NHS esters that would yield an elastic modulus of ∼ 1–2 kPa^48^ and a mesh size ξ of less than 10 nm after polymerization.^47^ From the evidence of surface conjugation via confocal microscopy and correlated polymer concentration, we anticipated that the PAAm characteristic mesh size in this study was less than 7 nm. As demonstrated in Fig. 2, while a green fluorescent protein (GFP) (MW ∼ 27 kDa, and R_g_ of 1.8 nm) diffused through a polymer network and was conjugated in the entire gel, an ITGB1 antibody, (MW ∼ 150 kDa) was conjugated exclusively at the peripheral region on the microgels. Maximum intensity projection confocal images revealed a distinct fluorescent signal at the periphery of the microgels when conjugated with ITGB1 antibody as compared to GFP, Figs. 2(c) and 2(d), respectively.

Collagens are the most abundant ECM proteins present in the body and the main component of connective tissue, thus have been widely used in cell adhesion and migration studies.^54^ In this study, type I collagen was conjugated exclusively on the periphery of the microgel particles Fig. 3(b). To visualize the COL1-LLS, we further incorporated rhodamine B acrylate (<0.1 mol. %) into the polymer backbone in addition to NHS ester groups [Fig. 3(a)]. This helps to confirm the microgel particles and the presence of conjugated type I collagen at the surface [Fig. 3(c)]. Since the conjugation process may induce changes in protein conformation, we cultured adherent glioblastoma cells (Kr158B) in the microgels for several days to test cell adhesion. As shown in Fig. 3(d), the cells formed network-like adhesion around microgel particles and acquired typical adherent morphology with extended actin filaments, demonstrating that the conjugated collagen proteins are functional postconjugation.

**FIG. 4.**
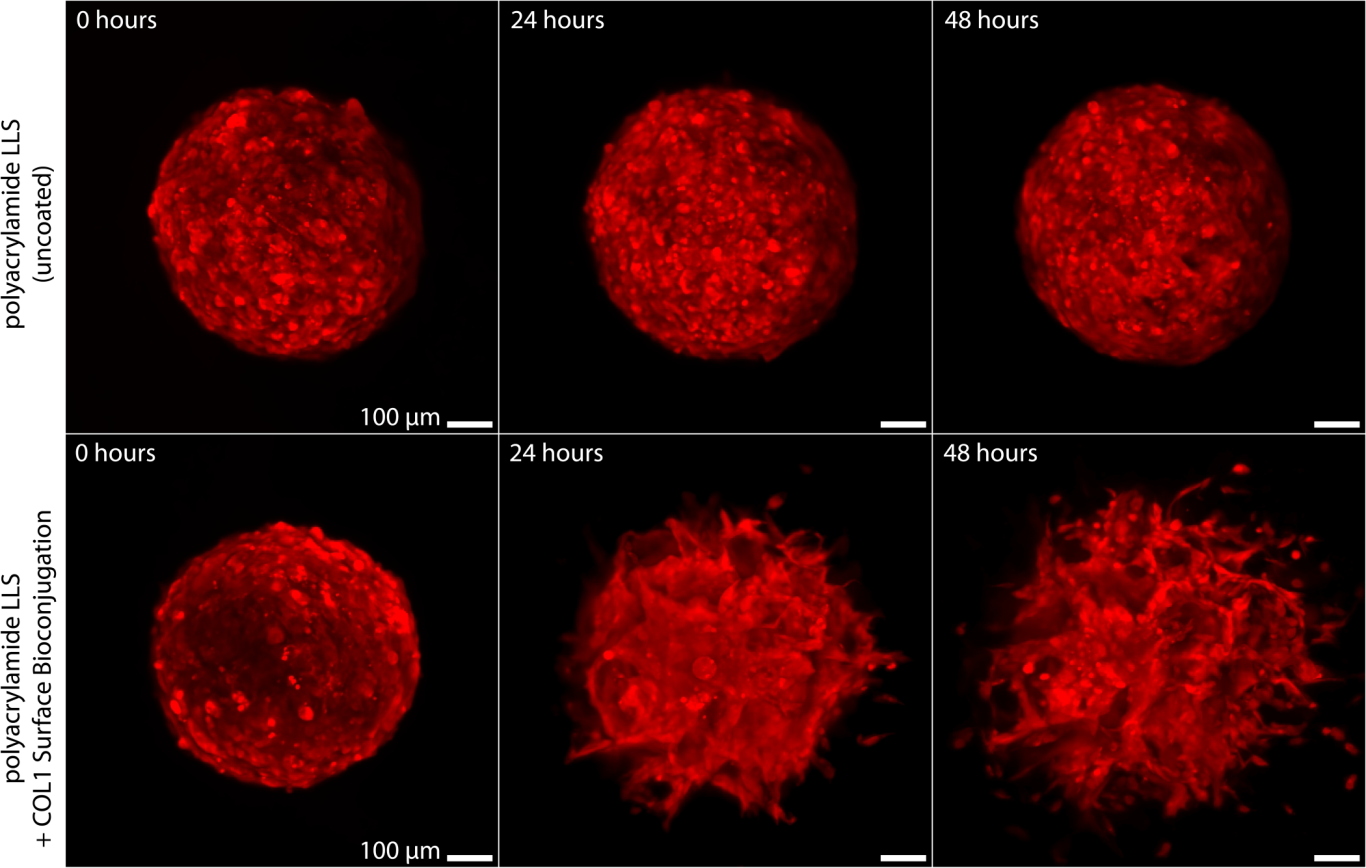
Glioblastoma invasion model on collagen-conjugated LLS—48-h confocal time-lapse images show the local 3D invasion of glioblastoma cells from a bulk cellular sphere cultured in inert LLS (top row) vs type I collagen (200 *μ*g/ml) conjugated LLS (COL1-LLS) (bottom row). The cells were stained for Cell Tracker Orange CMRA (see the videos in the supplementary material^75^).

In this study, we used a glioblastoma model to study invasion dynamics. As previously discussed, glioblastoma is the most aggressive cancer of the nervous system, and the tumor often grows and spreads rapidly.^55^ Glioblastoma tumors are highly invasive and have been reported to adopt an aggressive migratory pattern radially away from the tumor margin.^56^ In general, cancer cells from solid tumors are adherent but can become anchorage-independent survivors in low-adhesion substrates.^57^ In such environments, these cells interact with the neighboring cells and form aggregations for optimal survival. This mechanism has been commonly employed to develop in vitro 3D tumor spheroid models by various techniques (e.g., hanging drop^58^ and floating spheres^59^). In this study, the tumor spheroids were formed using a 3D platform for cell culture in LLS by means of perfusion as previously described.^34^ In short, cancer cells were cultured in inert LLS (without bioconjugation) using continuous directional perfusion. The platform ensures a constant supply of growth media and removal of metabolic waste. Due to the lack of adhesion on the surrounding substrate, cancer cells distributed within the inert LLS formed aggregates by day 2 and were grown into 400–600 *μ*m spheroids (∼day 7) (Fig. 4, top row). To investigate the invasion of tumor spheres, we cultured glioblastoma spheroids in COL1- and inert LLS. The tumor evolution was continuously monitored over time by *in situ* confocal imaging. The tumor spheroids, upon being cultured in COL1-LLS, demonstrated rapid interaction with their surroundings. The cancer cells at the tumor periphery adhered and migrated radially away from the tumor mass (Fig. 4, bottom row). Once the invasion initiated, the glioblastoma cells demonstrated both individual and collective modes of migration to rapidly infiltrate the surrounding.^75^

**FIG. 5.**
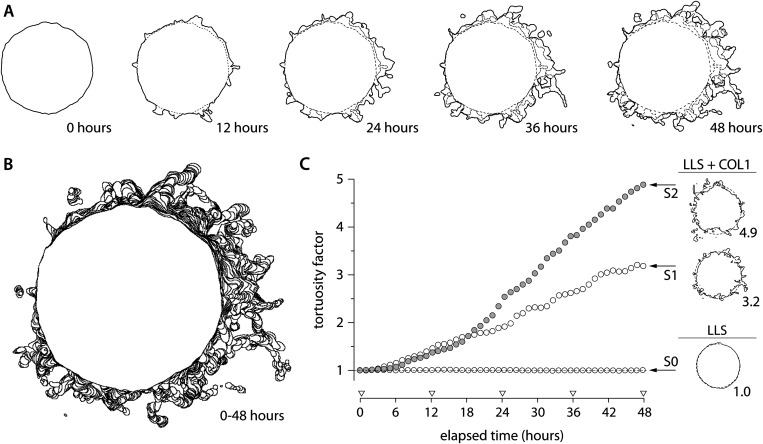
Tumor invasion as characterized by peripheral tortuosity. (a) Within 48 h, invasive fronts of a tumor rapidly protruded into the surrounding COL1-LLS in both individual and collective manners. (b) Peripheral cancer cells, at the interfacial contact with COL1-LLS, initiated opportunistic paths that led the way for the neighboring followers. The cohesive migration of cancer cells exploited paths of least resistance by navigating through interstitial space between the surrounding LLS particles. (c) In this study, the formation of invasive paths led to a notable distortion of the tumor periphery as characterized by a tortuosity factor. The factor describes the morphological characteristic of the tumor and its invasive phenotype potentiated by available adhesion molecules and the geometrical properties of accessible space in COL1-LLS.

Tumor morphology has been commonly used as a diagnostic factor for progression.^60^ Tumors with invasive phenotypes often carry significant mutational burdens, are highly proliferative with increased metabolic and oxygen consumption, demonstrate decreased cell-cell adhesion, and overproduce ECM-degrading enzymes.^8^ These factors stimulate cancer cells to become increasingly motile and interactive with the surrounding ECM. Various water-soluble (globular) and nonsoluble (fibrous) protein compositions of the ECM present different physical barriers and intrinsic adhesion molecules, thereby promoting heterogeneous modes of tumor invasion.^61^ In this study, we examined the nonenzymatic modes of migration by reconstructing the ECM from discrete blocks of collagen-conjugated LLS microgel ensembles. This platform allows for the local discrete stochastic process while ensuring a stable yield-stress property from a global continuum. The glioblastoma cells extended their filopodia (>10 *μ*m), probing for accessible pores, and established firm attachment to their surroundings (Fig. 4, bottom row and Fig. 5(a)]. Once properly adhered, leading cancer cells gradually pulled their body forward into the narrow space between COL1-LLS particles while maintaining appropriate distance with neighboring followers. The collective protrusions dramatically altered the tumor morphology over time, showing remarkably tortuous tumor margins [Fig. 5(b)]. We digitally analyzed tumor tortuosity by evaluating the ratio of 2D projected perimeters to the perimeter of a perfect circle of the same total internal pixel area. The characterization revealed that tumor spheres cultured in COL1-LLS medium (S1 and S2) had significantly higher tortuosity factors (>threefold), increasing monotonically over time, as compared to those cultured in inert LLS (S0) [Fig. 5(c)].

**FIG. 6.**
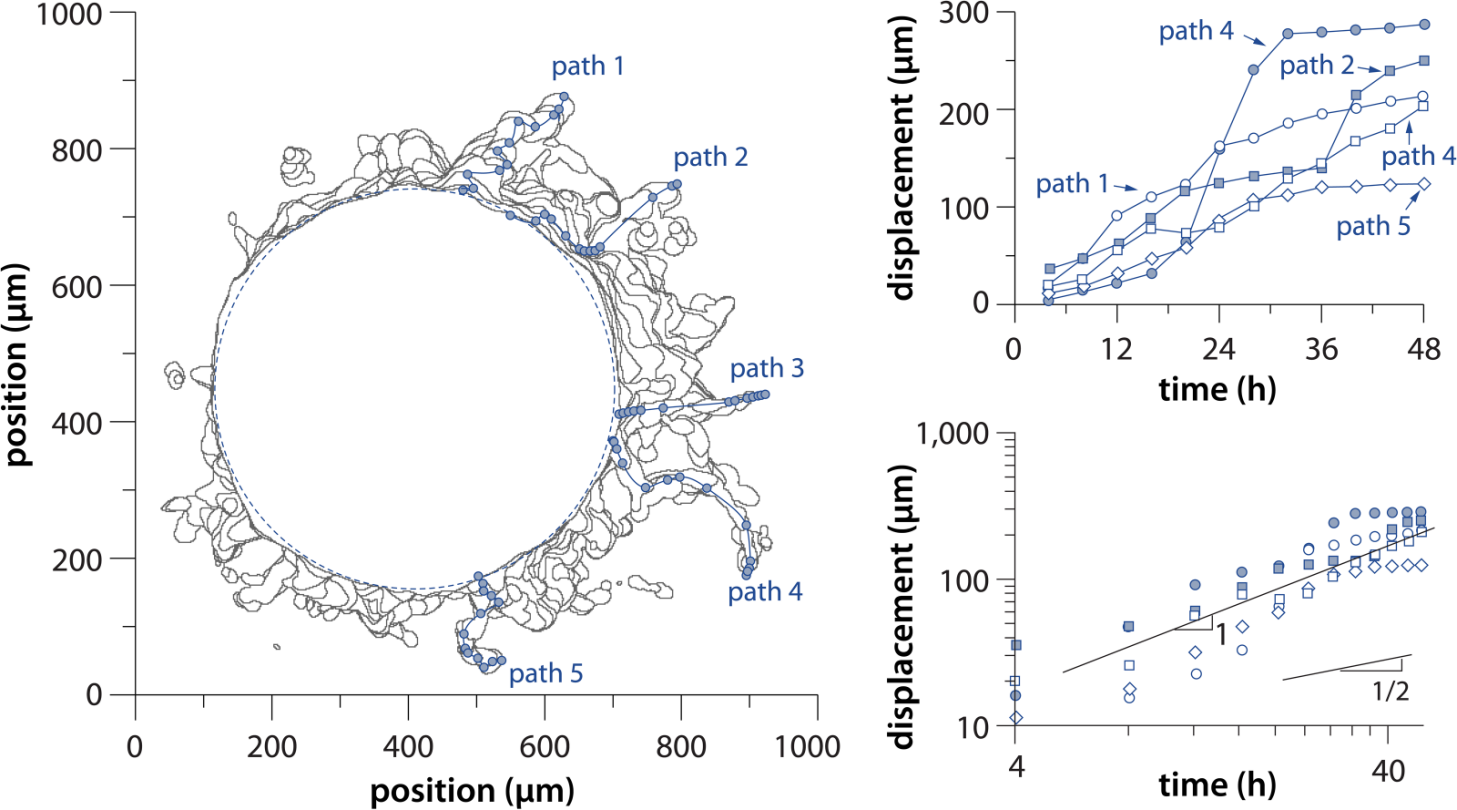
Path analysis of invasive fronts—illustration of the path analysis for five representative invasive projections of cancer fronts (paths 1–5) highlighted in blue. The displacement of the path fronts over time is plotted on a linear and log–log scale (right) demonstrating a super-diffusive phenomenon (slope ∼1 log–log bottom right). The data were collected in a COL1-LLS.

*In vivo* observations suggested that confinement and geometrical architecture of local TME can promote cancer invasion and metastasis.^21,24^ For instance, cancer cells can migrate along collagen fibers, demonstrating a sufficient mechanism for invasion.^11,16,62^ In another study, confined microchannels in microfluidic devices enable optimal cell contractility and rapid amoeboid migration.^24,25,63,64^ Here, COL1-LLS microgels imposed a 3D channel-like and random network of interstitial space presenting potential paths for cell migration. Despite the various paths of migration, the cancer cells invaded the COL1-LLS medium along predefined directions, independently and collectively in a leader-follower mode of migration.^17,61^ As shown in Fig. 6, the invasive patterns of cancer cells did not appear to be a randomly diffusive process. Instead, tracking the evolution of invasive paths revealed a super-diffusive (directional) behavior.

Since the LLS is made of polyacrylamide and is nondegradable, each microgel locally imposes a physical barrier to cell migration and forces cells to navigate around the tortuous and narrow network of interstitial space between the microgels. We hypothesized that the observed super-diffusive behavior is a result of cancer cells exploring and invading the three-dimensional space via preferential paths.^65–68^ To test this hypothesis, we performed off-lattice agent-based computer simulations of random walks in a 3D LLS network, which revealed super-diffusive behavior. We have also observed that the local invasion of different tumor models in the COL1-LLS is variable and regulated by several factors—the invasive phenotype, the gel mechanical property, and the size distribution of the LLS which modulates the interstitial space. The role of each parameter in migratory behavior has been investigated and will be reported in an independent study.^69^

## IV. CLOSING REMARKS

A new form of LLS with bioconjugation of COL1 has been described and shown to provide an accessible model to investigate cancer invasion. The *in vitro* model provides a unique opportunity to study the role of ECM proteins and biointerfaces on cancer invasion dynamics. Glioblastoma invasion is enabled by malignant geno/phenotypes under stress and can be facilitated by adhesion-dependent opportunistic migration into accessible spaces independent of proteolytic activity. Additionally, monotonically increasing tortuosity and super-diffusive behavior of glioblastoma invasion were measured in a COL1-LLS system.

Carcinogenesis and tumor progression occupy all three spatial dimensions, but the necessary infrastructure for establishing relevant 3D *in vitro* models has proved a significant challenge. In this study, we reconstructed the ECM component of a TME from a bioconjugated liquid-like solid as discrete units of physical support to study tumor invasion. Investigations on mechanisms of 3D cell migration have reported that anchorage-dependent phenotype is coupled with ECM degradation (MMP-dependent) while amoeboid migration (MMP-independent) is mainly enabled by cellular contraction and protrusion through accessible pores.^21,70–72^ We believe this study reports the first 3D tumor invasion model that decouples cellular adhesion and proteolytic capabilities. The bioconjugated LLS enables anchorage-dependent migration while the randomly interconnected microchannel network of the interstitial space provides paths of least resistance in all three spatial dimensions.

We envision the bioconjugated LLS platform will potentiate many applications to advance basic cancer research. For instance, we have employed the platform to evaluate CAR T cell locomotion in the solid tumor microenvironment and dissect the underlying immunosuppressive physical barriers of tumor–immune interaction.^73,74^ Furthermore, the LLS has enabled the long-term perfusion culture of cells and functional tissue microexplants in 3D.^34^ Here, the bioconjugated LLS will further allow formulations of ECM protein compositions for optimal *ex vivo* culture of delicate tissues. Together with perfusion culture, the ability to precisely control the transport of small molecules, drugs, antibodies, growth factors, and metabolites makes this system versatile with existing microbiology infrastructure, precise spatial organization for multiorgan-models, and integration with *in situ* high-resolution microscopy. The resulting system may afford the maintenance of patient-specific tissue microexplants with the inclusion of autologous immune cells and associated stromal components for personalized immunotherapy screening. Ultimately, the system is both a discovery platform and a testbed to facilitate the cross-fertilization of ideas, methods, approaches, and analysis techniques.

## Data Availability

The data that support the findings of this study are available from the corresponding author upon reasonable request.
